# Supporting families in end-of-life care and bereavement in the COVID-19 era

**DOI:** 10.1017/S1041610220000745

**Published:** 2020-04-30

**Authors:** K. J. Moore, E. L. Sampson, N. Kupeli, N. Davies

**Affiliations:** 1 Marie Curie Palliative Care Research Department, Division of Psychiatry, UCL, London W1T 7NF, UK; 2 Barnet, Enfield and Haringey Mental Health Liaison Service, North Middlesex University Hospital NHS Trust, London N18 1QX, UK; 3 Centre for Ageing Population Studies, Research Department of Primary Care and Population Health, UCL, London NW3 2PF, UK

## Introduction

Mortality from coronavirus disease 2019 (COVID-19) increases with age, and those over 80 are particularly vulnerable (Verity *et al.*, 2020). Most national data on COVID-19 will underestimate mortality in older people. Triage and resource allocation protocols (Truog *et al.*, 2020), and our understanding that it is often in the best interests of the frail older person to remain in their usual place of residence, may result in many deaths occurring outside of hospitals, particularly in care homes where these data may not be routinely collected.

However, the nature of COVID-19 is changing how people die. Patients can experience a sudden deterioration with rapid onset of respiratory failure. Frail older people and their families may have to make very quick decisions under highly stressful circumstances and decide whether to go to the hospital (where they risk iatrogenic harm, have proven worse outcomes, and will be separated from family and loved ones), or to remain at home, where they may be more comfortable. People residing in care homes may not be able to see visitors due to social distancing and other measures to restrict movement and contact outside the “household.” In addition, people who are at very high risk from COVID-19 because of severe preexisting health conditions may be required to “shield” (Kmietowicz, 2020) themselves and have severely restricted contact with family and friends.

UK government policy is to support vulnerable people, including frail older people and those with dementia, to make advance care plans. This is challenging as the person may lack the capacity to make decisions for themselves. Family carers may be required to act as proxy decision makers in their “best interests.” During the COVID-19 pandemic, families will need to make difficult decisions regarding resuscitation, treatment escalation, and place of care.

## End-of-life decision-making

The importance of discussing end-of-life care wishes with older people is highlighted even more so during a worldwide pandemic. Commonly, discussions about end-of-life care enable older people and their families to anticipate potential future challenges and to make plans accordingly. Topics include preferred place of care and death, Do Not Attempt Cardiopulmonary Resuscitation (DNACPR) orders, and ceiling of care and treatments such as avoiding hospitalization and declining antibiotic use at the end of life. End-of-life care discussions are encouraged with the aim to maximize comfort and avoid potentially futile and burdensome interventions at the end of life, increasing the quality of the dying experience for all.

Having discussions about end-of-life care can have multiple benefits. Significantly, it provides an opportunity for older persons to express their preferences for end-of-life care or to indicate who they would like to be involved in the decision-making process. This is important as they may be unable or lack capacity to contribute to these discussions when closer to end of life. There are also benefits for healthcare professionals, given that their confidence to discuss end-of-life care improves when they are equipped with knowledge of patient and family wishes (Poppe *et al.*, 2013). Similarly, when an older person living with dementia is involved in their end-of-life care discussions, carers experience less guilt and feel more prepared and confident implementing decisions at the end of life (Sellars *et al.*, 2019).

Guidelines generally recommend that these discussions take place during the early stages of a disease trajectory, in particular, in conditions where mental capacity may become compromised such as dementia (van der Steen *et al*., 2014). During the current COVID-19 pandemic, there are multiple reports that people have not planned their end-of-life care and not previously discussed with family their wishes and preferences should they become very unwell. Many older people including those with dementia are being encouraged to complete advance care plans (Curtis *et al.*, 2020); however, for some, this may already be too late as they lack capacity. Families may now be faced with new decisions including should they still go and visit their relative during the COVID-19 outbreak? This will be particularly difficult for those who live in a care home or have dementia and may be confused as to why their relatives no longer visit them. Families will be faced with making difficult and emotive decisions on their behalf as proxy. Many of these decisions, for example, about place of care and hospitalization, will need to be made rapidly in an acute situation.

During the pandemic, however, as death and dying become the focus of our day-to-day lives, the impetus for having these discussions and considering our end-of-life care preferences may be brought to the fore. Previous barriers, such as not perceiving death as imminent or difficulties anticipating the future (Dening *et al.*, 2013), may reduce. However, other challenges remain such as family carers lacking information to make informed decisions which they fear cannot be altered once made (Kupeli *et al.*, 2019) or not trusting healthcare professionals’ knowledge (Sellars *et al.*, 2019). Family carers may not have the time to process the information that is given to them to make informed choices or to discuss decisions with other family members such as siblings. Healthcare professionals may not have time or capacity to develop relationships with older people or family carers; most of whom they are unable to meet face to face prior to having these challenging and often difficult conversations. Professionals engaging in difficult conversations often rely on in-person and nonverbal cues to facilitate discussion of sensitive subjects; however, telephone conversations hinder this, and some people may not have access or the skills to use technologies such as video calling.

Families may be struggling to recall what they previously discussed with the individual, considering their previous wishes, and balancing that with would their wishes be the same in the current situation. However, for many where conversations have not occurred, they will be faced with uncertainty and trying to anticipate what they think the individual would want (Lamahewa *et al.*, 2018). In making decisions, families will be encouraged to consider the best interests of the individual. For many, these decisions will generate a wealth of emotional distress, anxiety, guilt, and burden (Caron *et al.*, 2005, Forbes *et al.*, 2000).

The substituted interests model is an approach that may help reduce the stress on family in end-of-life decision-making (Sulmasy and Snyder, 2010). Typically, decisions made by proxy decision makers are based on substituted judgments which require them to determine what the person lacking capacity would have chosen for themselves. However, studies have demonstrated that the preferences of proxies about end-of-life care do not always match with those of the older person (Harrison Dening *et al.*, 2016). The substituted interests model asks the proxy to describe the values of the person lacking capacity, such as cultural, religious, or personal preferences; important relationships; and who they would want to make decisions. The healthcare professional presents realistic information about the clinical circumstances and then draws on the values of the person to recommend a course of action that will be unique to that person (Sulmasy and Snyder, 2010). Decision-making becomes a joint process between the proxy and the healthcare professional.

During the pandemic, there has been a public rhetoric around DNACPR plans and hospitalization which may not be helpful. It is important to understand that this is not a binary decision but a conversation that opens up alternative and just as important types of care. Adopting a palliative approach is not denying care but means a person receives a more holistic and person-centered approach that is in keeping with their previous wishes and may maintain comfort, quality of life, and ensure a good death. Society as a whole has their part to play with this; supportive social networks and communities can help to desensitize these conversations and ensure conversations are discussed before acute events, better preparing everyone involved (Sawyer *et al.*, 2019).

Carers may benefit from support with making decisions (Mathew *et al.*, 2016) to better understand their options and the benefits of when a palliative approach may be appropriate. Decision aids which provide information on the decision and the options available have shown promise to support family carers (Davies *et al.*, 2019a), this may include novel approaches such as simple rules of thumb. Rules of thumb have been successfully used with professionals when making decisions about end-of-life care for people with dementia (Davies *et al.*, 2018). They break down complexity while ensuring not to oversimplify and lose the nuances and personal wishes which are important to consider. These approaches may be particularly relevant during COVID-19 when decisions have to be made rapidly, and there may be less support available from overstretched professionals and services.

## Grief and bereavement

COVID-19 will bring forward death for many older people. While grief is a normal part of life and death, circumstances around death can impact the grief process. Having discussions and being involved in decision-making are important elements for helping families and carers prepare for end of life. Feeling unprepared for death is associated with higher levels of complicated grief after death (Hebert *et al.*, 2006). In dementia care, family carers often start grieving before death as they experience a range of relationship and personal losses. Studies show between 47% and 71% of these carers report grief while caring and 20% experience complicated grief after death (Chan *et al.*, 2013). Carers may experience higher grief if they feel that the dying process was traumatic or unexpected and they may live with regretting care decisions or being unable to say goodbye to their family member (Supiano *et al.*, 2020). These scenarios may be heightened in this time of uncertainty and where families may not be able to see or have physical contact with their relative at the end of life. Families who are able to see a relative who is dying in hospital may then have to socially isolate afterward and not be able to attend the funeral. It is important to note that staff may also suffer from significant grief and moral distress following the deaths of their patients. They may have to give distressing news over the telephone or receive calls from multiple grieving relatives. This may be particularly traumatic for care home staff and other residents who will often consider their residents to be part of their family.

The three-tiered public health approach to bereavement suggests that most people will manage grief with support through their social network (Aoun *et al.*, 2015). Our recent work has shown that among a range of factors relating to preparation for end of life, social support had the greatest impact on grief while caring for a relative with dementia (Moore *et al.*, 2020). However, as we are confined to our homes, access to our social network may be reduced. Electronic and online forms of communication provide a useful avenue of support but may not be accessible to all (Davies *et al.*, 2019b).

“All societies have a death system with the basic functions of warning and prediction, prevention, caring for the dying, disposing of the dead, social consolidation after death, making sense of death, and killing” (Kastenbaum, 2008). In times of a contagious disease such as COVID-19, this system can be dismantled, and the cultural and religious rituals that help us process grief may also be stripped away. Funerals may be cancelled or attendees severely restricted. Their value in bringing together the social network to say goodbye and offer support may be lost. COVID-19 requires culturally appropriate and creative approaches to support family to remember and celebrate the life of the person who has died and to reduce the impact of the circumstances of death.

## Conclusion

The COVID-19 pandemic may help stimulate discussions regarding people’s end-of-life care preferences and break down some of the stigma associated with these discussions. However, within this context, there are also new challenges and obstacles to overcome. Greater pressures on healthcare systems will mean access to scarce intensive care resources may not be available to all, particularly those who may be considered less likely to recover from a severe case of COVID-19. Discussions about end of life usually benefit from relationship building between healthcare professionals, patients, and families, but current circumstances may limit communication between all parties. Technology may offer alternative forms for communicating where social distancing is in place, but these options may be challenging or not possible for many frail older people and people with moderate to advanced dementia. Decision aids may offer a practical tool to help carers make difficult decisions as proxies for the family members.

As the death toll from COVID-19 rises, particularly among older people, the opportunities for social support and rituals around death have become limited. This will impact on how people process their grief and may have lasting consequences after the worst of the pandemic has passed. Community acknowledgement and support for grief will require new approaches within the context of the COVID-19 era.
